# Envisaging a global infrastructure to exploit the potential of digitised collections

**DOI:** 10.3897/BDJ.11.e109439

**Published:** 2023-11-30

**Authors:** Quentin Groom, Mathias Dillen, Wouter Addink, Arturo H. H. Ariño, Christian Bölling, Pierre Bonnet, Lorenzo Cecchi, Elizabeth R. Ellwood, Rui Figueira, Pierre-Yves Gagnier, Olwen M Grace, Anton Güntsch, Helen Hardy, Pieter Huybrechts, Roger Hyam, Alexis A. J. Joly, Vamsi Krishna Kommineni, Isabel Larridon, Laurence Livermore, Ricardo Jorge Lopes, Sofie Meeus, Jeremy A. Miller, Kenzo Milleville, Renato Panda, Marc Pignal, Jorrit Poelen, Blagoj Ristevski, Tim Robertson, Ana C Rufino, Joaquim Santos, Maarten Schermer, Ben Scott, Katja Chantre Seltmann, Heliana Teixeira, Maarten Trekels, Jitendra Gaikwad

**Affiliations:** 1 Meise Botanic Garden, Meise, Belgium Meise Botanic Garden Meise Belgium; 2 Naturalis Biodiversity Center, Leiden, Netherlands Naturalis Biodiversity Center Leiden Netherlands; 3 Distributed System of Scientific Collections - DiSSCo, Leiden, Netherlands Distributed System of Scientific Collections - DiSSCo Leiden Netherlands; 4 University of Navarra, Pamplona, Spain University of Navarra Pamplona Spain; 5 Museum für Naturkunde Berlin, Berlin, Germany Museum für Naturkunde Berlin Berlin Germany; 6 UMR AMAP, CIRAD, Montpellier, France UMR AMAP, CIRAD Montpellier France; 7 Sezione di Botanica Filippo Parlatore, Museo di Storia Naturale, Università di Firenze, Via G. La Pira 4, 50121 Firenze, Italy Sezione di Botanica Filippo Parlatore, Museo di Storia Naturale, Università di Firenze, Via G. La Pira 4 50121 Firenze Italy; 8 iDigBio, Gainesville, United States of America iDigBio Gainesville United States of America; 9 CIBIO/InBio, Centro de Investigação em Biodiversidade e Recursos Genéticos, Universidade do Porto, Vairão, Portugal CIBIO/InBio, Centro de Investigação em Biodiversidade e Recursos Genéticos, Universidade do Porto Vairão Portugal; 10 CIBIO/InBio, Centro de Investigação em Biodiversidade e Recursos Genéticos, Instituto Superior de Agronomia, Universidade de Lisboa, Tapada da Ajuda, Lisboa, Portugal CIBIO/InBio, Centro de Investigação em Biodiversidade e Recursos Genéticos, Instituto Superior de Agronomia, Universidade de Lisboa, Tapada da Ajuda Lisboa Portugal; 11 Muséum national d'histoire naturelle, Paris, France Muséum national d'histoire naturelle Paris France; 12 Royal Botanic Garden Edinburgh, Edinburgh, United Kingdom Royal Botanic Garden Edinburgh Edinburgh United Kingdom; 13 Freie Universität Berlin, Botanic Garden and Botanical Museum Berlin, Berlin, Germany Freie Universität Berlin, Botanic Garden and Botanical Museum Berlin Berlin Germany; 14 Natural History Museum, London, United Kingdom Natural History Museum London United Kingdom; 15 Research Institute for Nature and Forest (INBO), Brussels, Belgium Research Institute for Nature and Forest (INBO) Brussels Belgium; 16 INRIA, Montpellier, France INRIA Montpellier France; 17 Friedrich Schiller University Jena, Jena, Germany Friedrich Schiller University Jena Jena Germany; 18 Max Planck Institute for Biogeochemistry, Jena, Germany Max Planck Institute for Biogeochemistry Jena Germany; 19 German Centre for Integrative Biodiversity Research (iDiv) Halle-Jena-Leipzig, Leipzig, Germany German Centre for Integrative Biodiversity Research (iDiv) Halle-Jena-Leipzig Leipzig Germany; 20 Royal Botanic Gardens, Kew, Richmond, England, United Kingdom Royal Botanic Gardens, Kew Richmond, England United Kingdom; 21 Ghent University, Gent, Belgium Ghent University Gent Belgium; 22 The Natural History Museum, London, United Kingdom The Natural History Museum London United Kingdom; 23 CIBIO, Centro de Investigação em Biodiversidade e Recursos Genéticos, InBIO Laboratório Associado, Vairão, Portugal CIBIO, Centro de Investigação em Biodiversidade e Recursos Genéticos, InBIO Laboratório Associado Vairão Portugal; 24 BIOPOLIS Program in Genomics, Biodiversity and Land Planning, CIBIO, Vairão, Portugal BIOPOLIS Program in Genomics, Biodiversity and Land Planning, CIBIO Vairão Portugal; 25 MHNC-UP, Natural History and Science Museum of the University of Porto, Porto, Portugal MHNC-UP, Natural History and Science Museum of the University of Porto Porto Portugal; 26 Plazi, Bern, Switzerland Plazi Bern Switzerland; 27 Ghent University, Ghent, Belgium Ghent University Ghent Belgium; 28 Ci2, Polytechnic Institute of Tomar, Tomar, Portugal Ci2, Polytechnic Institute of Tomar Tomar Portugal; 29 Centre for Informatics and Systems of the University of Coimbra (CISUC), Coimbra, Portugal Centre for Informatics and Systems of the University of Coimbra (CISUC) Coimbra Portugal; 30 MNHN, Paris, France, Metropolitan MNHN Paris France, Metropolitan; 31 Ronin Institute, Montclair, NJ, United States of America Ronin Institute Montclair, NJ United States of America; 32 Faculty of Information and Communication Technologies- Bitola, University “St. Kliment Ohridski”, Bitola, Macedonia Faculty of Information and Communication Technologies- Bitola, University “St. Kliment Ohridski” Bitola Macedonia; 33 Global Biodiversity Information Facility, Copenhagen, Denmark Global Biodiversity Information Facility Copenhagen Denmark; 34 Centre for Functional Ecology, Department of Life Sciences, University of Coimbra, Coimbra, Portugal Centre for Functional Ecology, Department of Life Sciences, University of Coimbra Coimbra Portugal; 35 Museu da Ciência da Universidade de Coimbra, Coimbra, Portugal Museu da Ciência da Universidade de Coimbra Coimbra Portugal; 36 Utrecht University, Utrecht, Netherlands Utrecht University Utrecht Netherlands; 37 Cheadle Center for Biodiversity and Ecological Restoration, University of California Santa Barbara, Santa Barbara, United States of America Cheadle Center for Biodiversity and Ecological Restoration, University of California Santa Barbara Santa Barbara United States of America; 38 CESAM & Department of Biology, University of Aveiro, Aveiro, Portugal CESAM & Department of Biology, University of Aveiro Aveiro Portugal

**Keywords:** machine learning, functional traits, species identification, biodiversity, specimens, computer vision

## Abstract

Tens of millions of images from biological collections have become available online over the last two decades. In parallel, there has been a dramatic increase in the capabilities of image analysis technologies, especially those involving machine learning and computer vision. While image analysis has become mainstream in consumer applications, it is still used only on an artisanal basis in the biological collections community, largely because the image corpora are dispersed. Yet, there is massive untapped potential for novel applications and research if images of collection objects could be made accessible in a single corpus. In this paper, we make the case for infrastructure that could support image analysis of collection objects. We show that such infrastructure is entirely feasible and well worth investing in.

## Introduction

Owing to their crucial role in documenting the Earth's biodiversity, global biological collections are likely to contain samples representing most known macro-biodiversity. These collections serve as invaluable assets for various research fields including ecology, conservation, natural history and epidemiology (Bradley et al. 2014, Davis et al. 2019, Antonelli et al. 2020). Furthermore, they represent an important yet underutilised resource for addressing global challenges (Soltis 2017, Hussein et al. 2022). They also play a role in the verifiability of research and, in some cases, the repeatability. Therefore, ensuring global access to these collections and integrating their data is of paramount importance for the future.

**Figure 1.**
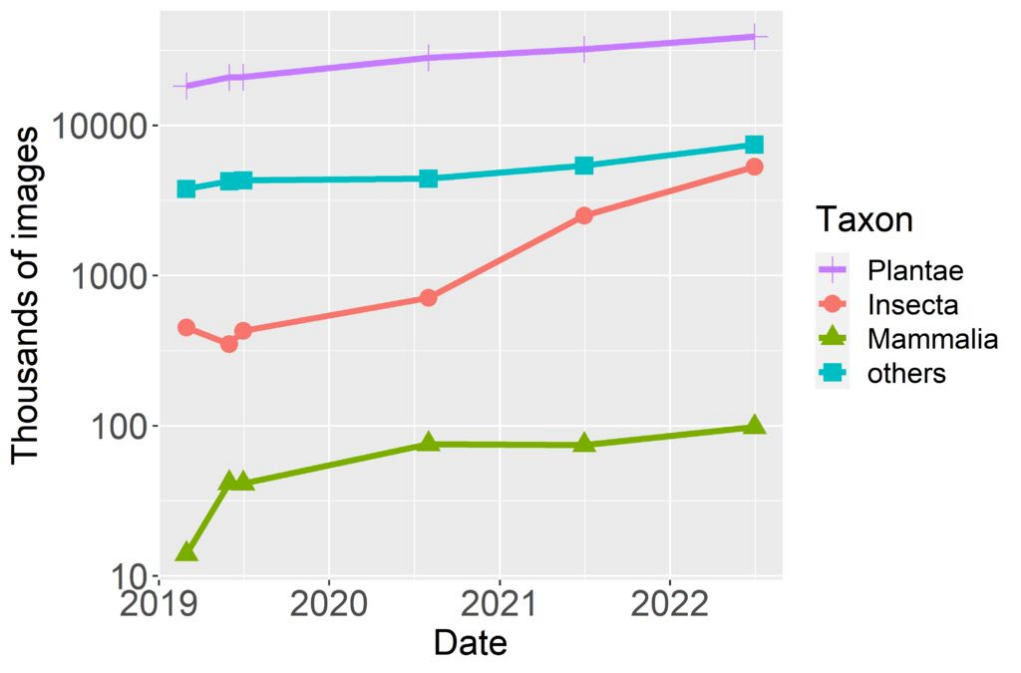
Progress in digitising natural history collections. A growing number of images are accessible from the Global Biodiversity Information Facility, iDigBio or BioCaSE. To examine the rate and volume of digitisation, we used six snapshots of these databases taken since 2019, using Preston, a biodiversity dataset tracker (Poelen 2022, Poelen and Groom 2022, Elliott et al. 2022). Although likely to be an underestimate of specimen images, because not all are linked to the snapshot datasets, trends give an indication of digitisation progress. The number of available images is increasing approximately exponentially. There are seven times more plant specimens than insects in our most recent snapshot, though insects are far more numerous in nature, an estimated 5.5 million species of insects (Stork 2018) vs. 350,000 plants (Cheek et al. 2020). Nevertheless, the rate of increase of insect images is faster and, if one extrapolates the curves, it is easy to imagine that insect images will surpass plant specimens in a few years. Imaging of mammalia (~ 6,400 species; Burgin et al. (2018)), while increasing, is not doing so as rapidly as insects.

To keep up with demand for access to collections, digital imaging of biological collections has progressed at pace (Fig. 1). In January 2023, the Global Biodiversity Information Facility (GBIF) had more than 51 million preserved or fossil specimens with an image. This number is expected to grow substantially. For example, digitisation of the Kew Herbarium, which holds over 7 million specimens, will add to already major digitisation programmes in Australia, China, Europe, USA and others (Nelson and Ellis 2018, Willis et al. 2018, Borsch et al. 2020, Chinese Virtual Herbarium 2021).

With this increase in digital images, it is not surprising that computer vision techniques are now being applied to them. In recent years, machine learning, in particular, has become mainstream and has been built into workflows that start with digital images and metadata and result in statements about what is shown. Such workflows can extract information about biological specimens from typed or handwritten labels (Allan et al. 2019). There are many other uses for image analysis of specimens, as we elaborate below (Pearson et al. 2020, Soltis et al. 2020).

Improving online access is important because collections are physically dispersed, yet interconnected (Nicolson et al. 2018). Researchers are rarely able to obtain a full set of specimens for a single taxon, collector or geography from a single institution. Most are scattered across tens or even hundreds of collections. Digital access breaks down physical barriers, making collections accessible as a unified research tool (Hardisty et al. 2020). Online collections data also serve as a resource for researchers who are not at institutions housing their specimens, a particularly important issue given historic imbalances in the amassing of collections in the Northern Hemisphere, from high-biodiversity regions elsewhere (Grace et al. 2021).

Unified access to specimen images is particularly important because image files are comparatively large and image analysis pipelines are demanding on processor time. Current internet bandwidth makes transferring large numbers of files a bottleneck, particularly if they need to be moved multiple times. Therefore, it makes sense to store large numbers of images close to where processing will occur. While such infrastructure exists for other data types (e.g. *Copernicus* for remote sensing and *WLCG* for the Large Hadron Collider), no such support exists for biological collections-based image processing. Researchers amass images and process them independently, which is unscalable and is unsuitable for dynamic image corpora and workflows intended to run multiple times.

**The Vision**

We envisage a data space for biological collections with a centrally accessible image corpus with built-in processing. This will allow anyone to access digitised images of specimens, without having to concentrate on the logistics of corpus creation and maintenance. Building accessible interfaces would also remove technological barriers that prevent taxonomists, ecologists and others from using advanced analysis tools. Through supervised expert contributions, the system could integrate knowledge from many disciplines. Such a corpus would constantly be furnished with new images from publishing collections and support citation and reproducibility of workflows and their underlying collections, in alignment with FAIR Data Principles (Wilkinson et al. 2016). It would make it easier to curate image datasets and use them for research (e.g. for benchmarking and challenges for machine learning) and for activities like teaching species identification.

**The Scope**

Images from living organisms are not considered here, nor other media, such as sounds, though they are undoubtedly useful and deserve attention. Though the Al challenges of images of living organisms are different, their numbers are at least two orders of magnitude larger and increasing more rapidly than digitised preserved specimens and dedicated infrastructures already exist to process them, such as Pl@ntNet and iNaturalist. The creators of such images are also more varied, as are the relevant licensing requirements. An exception might be images of living organisms *in situ* before they were preserved. Such images give additional context to the specimen and can potentially be used alongside the preserved specimen for human and computational comparison (Goëau et al. 2021).

In this paper, we present the purposes for a unified infrastructure of specimen images and envisage what it might look like. We answer the questions: what research could be done with such an infrastructure, who would use it, what functionality would be needed and what are the architectural requirements?

We imagine a future where we can search across global collections for such things as the pattern of a butterfly’s wing, the shape of a leaf, the logo of a specific collection, or for examples of someone’s handwriting.

## Purposes

Infrastructure needs to justify its costs through benefits, not just for science, but wider society. We also need to understand the users and other beneficiaries. Below, we outline some uses and users for an imaging infrastructure for collections; there are undoubtedly more we have yet to imagine.

**Species identification**

Most experiments with species identification from specimen images have focused on herbarium specimens (Carranza-Rojas et al. 2017, Kho et al. 2018, Pryer et al. 2020, Hussein et al. 2022). This is because they are two-dimensional, follow a fairly standardised format and are highly available. Digitisation of herbarium specimens has preceded that of other organisms. Nevertheless, because insect specimens (Fig. 1), in particular, are much more numerous, there is clear demand for their automated identification (Valan et al. 2019, Høye et al. 2021). Insect colour and morphology are well preserved in specimens. This means that automatic identification trained on specimens may work on living insects and vice versa, having the possibility to create training datasets for rarely-seen organisms (Goëau et al. 2021, Goëau et al. 2022). Specimens from natural history collections have also been used successfully to train models that assist in sorting images from camera traps deployed in ecological monitoring (Høye et al. 2021).

The state of preservation, uniformity and distinctiveness of pollen grains also makes them good targets for automated identification, whether they are from preserved collections or fresh. Indeed, pollen is well preserved as fossils and sub-fossils making them useful targets to analyse evolutionary and ecological change (Romero et al. 2020, Hornick et al. 2022). Machine learning could transform pollen identification into a much more routine process (Bourel et al. 2020), with potential applications in environmental monitoring, archaeology and forensics.

The main advantage of automated identification of digital images of preserved specimens is not accuracy, but potential for high throughput. Accessing large numbers of images in a suitable computational environment remains a critical factor to mainstreaming automatic specimen identification across collections.

**Extracting trait data**

Morphological, phenological and colourimetric traits are often visible on specimen images (e.g. Fig. 2a). Such traits might be diagnostic and are also used to understand how traits evolve and what they tell us about evolution. Some animals, such as insects and birds, maintain colour well and may be interesting targets for research (Hoyal Cuthill et al. 2019, Hantak et al. 2022). Amongst other avenues, studies have shown that colour is an important factor in climate change adaptation of insects (Zeuss et al. 2014).

**Functional traits**

Morphological functional traits have been used to predict impacts of climate change on ecosystem functioning (Pigot et al. 2020), species distributions (Pollock et al. 2011, Regos et al. 2019), community structure (Li et al. 2015) and how these traits fit into the land surface component of climate models (Kala et al. 2016). Functional traits recorded from preserved specimens supplement field-recorded data, filling geographic and temporal gaps and providing legacy data (Heberling and Isaac 2017, Bauters et al. 2020, Kommineni et al. 2021), as well as potentially enabling discovery of newly-relevant morphological traits. Examining such traits in preserved specimens is considerably cheaper than fieldwork.

Leaf morphological traits are particularly amenable to extraction from herbarium sheets, because they are laid flat and do not necessarily require magnification (Heberling 2022). Size, dimensions, arrangement, dentation and venation are all targets for machine learning and experiments with extracting these parameters have shown it to be feasible and reliable (Triki et al. 2020, Weaver et al. 2020, Heberling 2022, Weaver and Smith 2023). Extraction of traits from collections of insects has great potential as their state of preservation is high (Høye et al. 2021).

In the case of fish, the large number of species globally, enormous number of morphological traits and substantial variation mean we can only hope to fill gaps in our knowledge of traits if preserved specimens are used (Hay et al. 2020). Furthermore, specimens have the advantage that there is a voucher where measurements can be verified and new measurements taken.

Using well-documented algorithms for extracting traits from specimens would be much more efficient if a single large corpus were available for analysis and measurements could be less prone to error and more reproducible if source code and training data are open and shared (Meeus et al. 2020).

Collection practices have changed considerably over more than four centuries (Kozlov et al. 2021). Additionally, characters of specimens can change upon preservation, for instance, shrinkage associated with drying (Tomaszewski and Górzkowska 2016). Yet, with suitable awareness and controls, there is much to be learned from trait data gathered from digital specimens.

**Phenology**

A trait of particular interest for climate change studies is phenology. Changes in seasonal temperatures and rainfall affect hatching or emergence of dormant animals and maturation of leaves, flowers and fruits. Such changes may lead to a mismatch in seasonality amongst organisms (Renner and Zohner 2018). Detecting the phenological state of an organism is possible through machine learning (Lorieul et al. 2019, Davis et al. 2020, Triki et al. 2021, Goëau et al. 2022, Katal et al. 2022) though not to the level of accuracy achieved manually. Nevertheless, the obvious advantage of machine learning is the potential for high throughput processing of images to track phenological shifts (Pearson et al. 2020).

**Species interactions**

Organisms are in constant conflict with predators, parasites and pathogens. Specimens provide a record of this, revealing long-term changes related to environmental change, such as the introduction of non-native species (Vega et al. 2019), pollution and climate change (Lang et al. 2019). For example, manually-extracted changes in leaf herbivory of herbarium specimens were correlated with climate change and urbanisation in north-eastern USA (Meineke et al. 2019). Meineke et al. (2020) further investigated the potential for extracting leaf damage data from herbarium specimens, through detection and classification of images split into grid cells.

**Collections care, curation and management**

Information is also needed for curation, organisation, storage and management of collections. An example is the need to identify specimens treated with toxic substances, such as mercuric chloride formally used to prevent insect damage. Over time, mercuric chloride leaves stains on mounting paper. Schuettpelz et al. (2017) used a convolutional neural network to detect such stained sheets. It has a false-negative rate of 8%, which is comparatively high error for a situation related to toxicity, yet could likely be improved with provenance information.

One can imagine image analysis workflows that detect the type of mounting strategy and preservation state of specimens. This would help curators triage remounting or other forms of curational care.

**Visual features of the specimen**

**Image segmentation and object separation**

Image segmentation is a fundamental image-processing task to facilitate higher-level tasks, such as object detection and recognition (de la Hidalga et al. 2022). In preparation for analysis, such as searching for signatures or to support a human-in-the-loop, it is often more efficient to recognise individual objects in an image, classify them and separate them into multiple images - for example, if images contain multiple specimens or labels need to be extracted for transcribing. Specimens from different collections show variety in backgrounds, caused by different mounting techniques and digitisation processes. Separating objects in preparation for further analysis may help establish training sets that ignore differences in background and positioning.

In an infrastructure built for image analysis, standard segmentation workflows could be run and optimised to avoid researchers repeating these steps and users could choose whether to analyse the whole image, all segments or specific classes of segment.

**Labels**

Specimens are usually annotated with information on labels. In the case of plants, these labels are on the mounting paper; for insects, they are on the mounting pin; while for larger zoological and plant specimens, labels might be tied to the specimen or on, or in, specimen jars. Therefore, as images of specimens often contain text, it is useful to provide printed and handwritten text recognition as part of an image processing pipeline. If text can be recognised, these additional metadata can be used to enrich items of the collection and automatically perform cross-collection linking. Furthermore, recognised text can aid in the digitisation process and validation of metadata, reducing manual input and improving data quality (Drinkwater et al. 2014).

Although state-of-the-art text recognition performs well on printed text, accurately recognising handwritten text is still a challenge. Older handwritten text might contain unique style, but even such cases can still provide valuable information, for example, text written by the same author could be automatically clustered, based on visual similarity and used to identify the collection and reduce manual validation.

**Figure 4.**
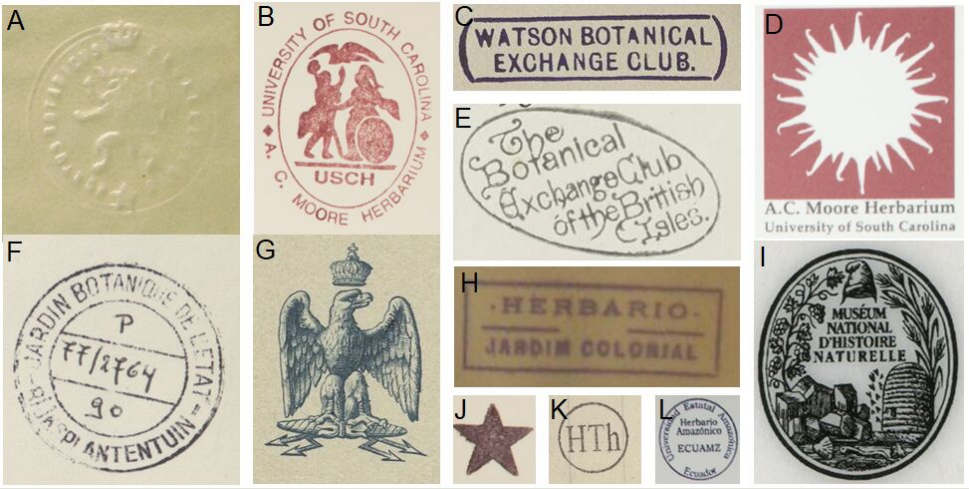
Embossed crests and stamps on herbarium specimens. **A** Lion and crown signifying ownership by the Botanical Garden of Brussels BR0000013433048 of BR Herbarium (CC-BY-SA 4.0). **B** Stamp of the A.C. Moore Herbarium at the University of South Carolina as on specimen USCH0030719 (image in public domain). **C** Stamp of the Watson Botanical Exchange Club on specimen E00809288 of the Royal Botanic Garden Edinburgh Herbarium (public domain). **D** Stamp of the A. C. Moore Herbarium at the University of South Carolina, USCH0030719 (public domain). **E** Stamp of the Botanical Exchange Club of the British Isles on specimen E00919066 of the Royal Botanic Garden Edinburgh Herbarium (public domain). **F** Stamp with handwriting is evidence of a loan from the BR Herbarium to the Herbarium Musei Parisiensis, P, on specimen BR0000017682725 of Meise Botanic Garden (CC-BY-SA 4.0). **G** Printed crest, P00605317 held by Museum National d’Histoire Naturelle (CC-BY 4.0). **H** A stamp on specimen LISC036829 held by the LISC Herbarium of the Instituto de Investigação Científica Tropical. **l** a crest used by the Muséum National d’Histoire Naturelle (MNHN - Paris), on specimen PC0702930. (licensed under CC-By 4.0). **J** A stamped star with unknown meaning on the same specimen as (B). **K** A stamp belonging to the Herbarium I. Thériot, on specimen PC0702930 at the Herbarium of the Muséum National d’Histoire Naturelle. (CC-BY 4.0). **L** A stamp belonging to the Universidad Estatal Amazónica, now housed in the Missouri Botanical Garden Herbarium under catalogue number 101178648 (CC-BY-SA 4.0).

Besides text, secondary data hidden in handwriting, ink colour, mounting paper, label shape and printed label decorations (Figs 2, 3, 4) can be used to determine their origins and history. Image analysis by itself can be enough to cluster specimens for particular purposes, for example, a group from a particular expedition.

**Rulers and colour checkers**

Another element often seen on digitised specimen images are rulers, scale bars and colour checkers. These are very varied, for example, in size, often customised for particular imaging campaigns. Colour checkers are used to validate colour fidelity of specimen images, while rulers provide a reference to the actual specimen size. Especially when digitising with a digital camera, it can be complex to calculate the actual dimensions of the specimen, as it depends on the lens and individual camera parameters. Therefore, detection of rulers and colour checkers on digital images can prove useful to estimate the actual sizes and correct colour balance. A generic object detection or instance segmentation model can be trained to detect these common objects. If all rulers in a collection are of a fixed size, the length of the detected ruler can be used to calculate a transformation from pixels to the ruler’s unit of measurement (e.g. cm, mm). This can then be combined with specimen segmentation models, to automatically extract dimensions and specimen traits (Triki et al. 2021). When rulers are not of uniform size, the distance transformation needs to be estimated by calculating the pixel distance between the measurement stripes or bars on the ruler (Bhalerao and Reynolds 2014). To extract the specific unit of measurement, the text denoting the unit on the ruler can be recognised or additional metadata about the specimen can be used to infer it.

**Finding stamps and signatures**

Specimens are often stamped, printed or embossed with crests that indicate provenance or ownership (Fig. 4). An example are those of botanical exchange clubs (Fig. 4), which operated in Europe from the middle of the 19^th^ century into the 1930s (Groom et al. 2014). Tens of thousands of specimens were exchanged this way. If a specimen was part of a botanical exchange club, it implies that duplicates exist and circumscribes the dates of collection. Although stamps usually contain some text, they are often not easily read with standard OCR engines.

Many specimens are signed, either by their collector, determiner or both (Figs 2, 3). Expert curators within an institution learn to recognise signatures of prolific collectors, but they are often illegible without that knowledge. Yet, it is common practice to use the name of a collector, together with their collecting number to identify a collection event uniquely. Furthermore, due to exchanges, loans and gifts, a collector’s specimens may be spread amongst a number of institutions. If the name is not distinct enough to be transcribed accurately, finding the specimens from a specific collector across the whole corpus of global collections would be an impossible task without some automated process.

**Unsupervised learning**

The stacked layers of deep neural networks can be regarded as a set of transformations that learn useful representations of the starting data. Using representations of specimen images learned by neural networks, rather than extracted metadata, would allow content-based interaction with and comparison between images. Such interaction is useful for tasks where a high-quality labelled dataset does not currently exist or where the characteristics of a specimen that are important to a task are not well-defined. For instance, White et al. (2019) used representations of specimen images learned by a neural network trained to classify fern genera to directly compare specimen morphology and test biogeographic hypotheses. Similarly, Hoyal Cuthill et al. (2019) trained a network to estimate the similarity of two sets of butterfly specimen images and used the learned representations to test mimicry hypotheses.

Some tasks require researchers to inspect and compare specimen images individually. The reduced dimensionality of deep representations in combination with scalable nearest-neighbour search (Johnson et al. 2021) makes direct comparison of images very efficient. This opens opportunities to explore collections through image content rather than through metadata and makes it possible to search a collection for similar specimens during identification and identify misidentified or poor-quality specimens.

Recently, interest in learning useful representations from unlabelled data has surged (Rives et al. 2021) in the field of unsupervised (or self-supervised) representation learning. These studies have shown that large numbers of unlabelled images (millions to billions) can be used to learn representations that work well as a starting point for supervised classification tasks, such as species identification (Walker et al. 2022). A large repository of images would facilitate this research by allowing the development and curation of the two types of dataset necessary for self-supervised representation learning: large training corpora and smaller, task-specific benchmarking datasets (Van Horn et al. 2021).

## Conceptual Framework of the Infrastructure

**Figure 5.**
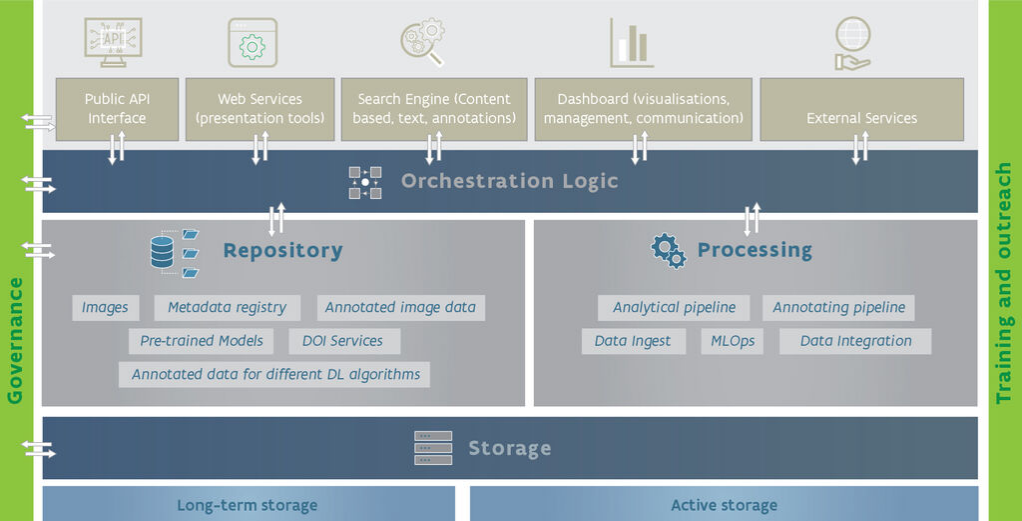
Framework of an infrastructure for analysis of specimen images showing the services, storage and relationships between them.

Unlocking the potential for machine learning in natural history collections is contingent on technical infrastructure which is easy-to-use, interoperable with regional and global biodiversity data platforms and accessible to the global scientific community. Here, we present a conceptual framework conceived as a roadmap for building such infrastructure. Although the infrastructure could be implemented in different ways (e.g. distributed or centralised), we describe three core technical components, coordinated by the orchestration logic: (1) the repository to index data and metadata; (2) the storage of images, models and data; and (3) the processing of images to generate new data, annotations and models (Fig. 5). The orchestration logic will consist of components such as technical workflows, security protocols and application integrations that enable implementation of business logic and access to services. In addition to technical components, the infrastructure will require a governance structure and set of protocols, as well as training and outreach to reach the intended audience.

**Component 1: The Repository**

A dedicated repository is needed which will reference and index information, such as specimen metadata, image metadata and annotations, alongside machine-learning models with their performance metrics and outputs (Fig. 5). Some existing infrastructures partially accommodate these data types, such as GBIF for specimen data, but none integrates the full spectrum of specimens, images, models and model outputs. These existing infrastructures can be reused, either by integrating or connecting with the repository or becoming it by extending their own capabilities. The repository should operate on FAIR principles, facilitating data discovery and reuse. This includes support for, or provision of, persistent identifiers for the different types of content, as well as different data standards.

Image metadata in the repository will include a reference to the image object located in the storage layer (Component 2), along with annotated training image data. Different kinds of image annotations will be supported, including geometric-based regions of interest (ROI), taxonomic or ecological traits and textual representations of label data. For interoperability, data standards supporting machine readability of these annotations are required. As different standards exist for these annotations and not all are equally suitable for any model, the platform should ensure support for multiple standards, such as *COCO* (JSON), *Pascal VOC* (XML) and image masks (rasterised or vectorised images). Multiple annotations can be made on a single specimen record, making persistent record identifiers vital. Metadata indexed in the repository will facilitate findability of suitable annotations, for instance, to serve as training data. A feedback mechanism may be implemented to correct and/or update annotations.

Pre-trained machine-learning models will be stored in the repository and made available for reuse, along with accuracy metrics and model outputs, such as segmented features or species metadata. To ensure findability, models should be classified by use-case through the use of keywords, since they are often trained for very specific use-cases, but could later be reused in other contexts. As part of the metadata, suitability scores will facilitate comparison of models in terms of their efficacy, possibly through community feedback or by analytics that take standardised model performance metrics into account. These results should be linked to the original images used in the training of the model (on the platform) and also to the images that were analysed in the use case. Some of this might be achieve by implementing the International Image Interoperability Framework (IIIF); for example, a IIIF compliant server could provide the segments of images dynamically (Snydman et al. 2015).

Persistent identifiers, such as Digital Object Identifiers (DOIs) or hash-based content identification (e.g. *Software Heritage PIDs* for code or simple SHA-256 hashes for images), will be assigned to digital objects produced during the use of the infrastructure, to make them citable. It will also be possible to assign persistent identifiers to versions, reflecting any subsequent updates to the digital objects. The repository will display citations of the persistent identifiers, including links to publications in which they are included, as well as any instances of their reuse in other projects within the repository. It is not only important to make the digital objects or outcomes openly available, but also under appropriate licences (e.g. *Creative Commons*) as indicated by the *FAIR for research software (FAIR4RS) working group* and Labastida and Margoni (2020). In many cases, a CC0 licence waiver would be appropriate, because of the lack of a novel intellectual creation step (Patterson et al. 2014, Egloff et al. 2017).

Managed through the orchestration logic, the repository is connected to a storage system and the processing unit, while having features, such as a content-based search engine, to browse not only on the traditional human-annotated metadata (e.g. date and place of observation, taxonomy and others), but also on information extracted from the images themselves. Advanced features can be built into the system, such as the ability for users to upload an image and search the catalogue by similarity (e.g. similar handwritten signatures) or query and filter collections of data using indexed metadata extracted from observations, either humanly or automatically annotated. In general terms, such functionality can be summarised as the ability to aggregate to each specimen media record all the information that is extracted from it either manually or automatically and indexed making it available to query.

Some good examples of similar content-based systems exist in production today. *Pl@ntNet*, *BeeMachine* and *iNaturalist* provide species identification of living organisms from photographs. Results can be refined by providing the user’s location, limiting possible results to the most likely matches. A more general example is Google Image Search, where anyone can search images using either a keyword (e.g. dog) or using an image as the search term. This function is also available on Google Photos, where a user can search their personal photos for specific people, different types of objects, places, ceremonies and so on. Although different, all those systems share similar logic: (1) they include models trained for specific tasks (e.g. object detection) that have been created offline using massive datasets in large GPU clusters (e.g. *Model Zoo* and *COCO dataset*); (2) when a new image is added to the collection (or possibly all, when new models are deployed), in addition to the submitted user tags, the images are processed with these models (inference/prediction pipeline) and tags are extracted; (3) the extracted information is saved and indexed and made available as searchable data. The envisioned system should provide similar functionality, with the added complexity of the myriad of different models and images illustrated by the use cases in the previous section.

**Component 2: The Storage**

The storage component (Fig. 5) encompasses all physical storage that is a local part of the platform and on which images, models, metadata and results are stored. It also includes functions, managed via orchestration logic, required to manage those data as far as access control (e.g. governance) and low-level file management is concerned (such as back-ups). Higher level management, such as handling uploads, selection of specific images and the moving of images to processing, is the responsibility of other components. The storage component is divided into two areas, archive and regular (active) storage. This distinction is primarily a technical one, separating high-performance storage required for accessing images while training models, from less advanced storage for other purposes.

Whether images are mirrored from their original source on to the platform or only downloaded temporarily on to the platform when needed, is a technical design question that should be answered during implementation. While this choice has no functional impact, it does have profound technical implications, as well as budgetary consequences. Locally mirroring all images referenced in the repository guarantees availability and predictable speed of access, but will also require extensive management to accurately reflect changes made to the source material and will take up an increasingly large storage volume. On the other hand, while downloading images on-the-fly greatly diminishes the required storage volume, it implies less control over availability and carries the risk of images becoming unavailable over time.

Scientists are already used to large communal storage infrasturecures, such as Dryad and Zenodo. Zenodo was developed under the European Organisation for Nuclear Research (CERN) and supports open science by providing a platform for researchers to share and archive their data and other research outputs.

**Storage of training images**

Images to use in training are discovered through the repository component, which functions as a central index of images, metadata, models and results. Actual image files might be hosted on the platform, or remotely, on servers of associated parties. In case of the latter, because of the technical requirements (i.e. high throughput, guaranteed availability, low latency), these images must be downloaded to the platform and be made available locally to be used in the training of models. Image selection is done in the repository and the orchestration logic functions as a broker between the repository and remote hosting facilities, taking care of downloading images. The storage component is responsible for the local storage of these files. This includes facilitating access control (i.e. keeping track of what images belong with which training jobs) and making images available to the processing component, where the actual training takes place. In the scenario where the local storage of training images is temporary, the images will be deleted once the training cycle of a model has been completed, while only the references in the repository to those images are retained with the resulting model. The handling of images while stored in the system, including their accessibility and deletion policies, is subordinate to the platform’s governance policies.

**Storage of models**

Once a model is deemed suitable for use, it may be published as such in the repository. The repository functions as a central index that allows researchers to find suitable models, while the actual code that makes up a model will be stored in the storage component. Once a model has been selected for use (see also next section), it is retrieved from storage and copied to the processing component. A similar scenario applies when a stored model is used as the basis from which to further train a new model or a new version of the same model (transfer learning). Since there are no specific performance requirements for storing a model, they will be stored in the archive section of the media storage component. Besides models that have been trained locally, the platform can also host and publish models that were trained elsewhere. From the point of view of storage, these models are treated as identical to ones trained locally. As with images, availability of and access to models stored on the platform is subject to governance policies.

**Storage of images for analysis**

Another function of the processing component is using ‘finished’ models for image analysis, resulting in annotation of newly-uploaded images with or without metadata (such as classification or identified regions of interest). For this purpose, images will be uploaded by researchers, after having selected a model or models from the repository to run on the images. Uploaded images will be stored in the storage component and kept there for the duration of the experiment. Responsibility for running these experiments, including the loading and execution of the selected models, lies with the processing component. Actively making available the images to the models is facilitated by orchestration logic.

Once experiments have been completed, these images will be moved to a low-performance part of the media storage component (archive storage), where they are stored with the newly-acquired metadata, in line with relevant governance policies. These archived images and their annotations are registered in the repository component, so as to make them findable. If, at a later stage, someone wants to perform further analysis on them, these images can be moved back to the active storage area.

The technical requirements for analysis processes are far less demanding than those of training processes, especially with regards to the need for constant high throughput. It is, therefore, conceivable that the platform will allow access to stored models through an API, in which case no images are stored locally.

**Storage of model results**

User value is gained from access to results derived from the models on the platform. These results might be produced as described hitherto or by use of a model remotely, either via API access or even by entirely running a model remotely. The form of these results can be manifold; besides previously mentioned examples, such as classification or the identification of regions of interest, they can also include more generalised performance characteristics of a model, such as the average recall and precision for a given set of images in case of a classification experiment. Uploading such results, in whatever format they might take and associating them with the models that generated them is the responsibility of the repository component, while the physical storage of data is taken care of by the storage component. Negotiation between the two components, both when storing and when retrieving, is performed by the orchestration logic. Again, all handling of these results follows the platform’s governance policies.

**Component 3: The Processing**

The processing component encompasses all the services and pipelines to compute tasks on batches of data, incoming or already existing in the system, such as those stored in the repository and storage components (Fig. 5). In other words, it supports a myriad of computational-intensive tasks, from ingesting new data, to the automated extraction of information from media, as well as exporting new datasets or scheduling the training of new models or the retraining of old ones.

This component requires a considerable amount of computing power to handle all the scheduled tasks in the system, which can even be elastic (i.e. cloud principles) given the fluctuating demand. These are delegated by the orchestration logic component, a set of services that are responsible for handling external requests, such as those from front-end applications or other external services using public APIs, serving as both gateway and manager to the main internal components – repository, storage and processing (Fig. 5). The greatest computational demand comes from tasks related to the creation of models, periodically updating the existing services or adding new ones. For these, specific hardware capabilities, such as several GPU/TPU instances, may be required from time to time.

The processing component and the tasks and services supporting it, should be able to scale vertically, that is, to handle more tasks by adding more RAM, more CPU cores or a better GPU to a cluster node, but preferentially also able to scale horizontally, namely, by adding more nodes, hence able to process multiple independent tasks in parallel.

The processing component can be organised into sub-components, amongst which are: (1) Data ingestion; (2) Machine-learning models and analytics services (such as image segmentation, objection detection and image classification); (3) Analytics pipelines (processes or programming scripts built to provide analytical services); (4) Data integration; and (5) Data export, which helps to deal with any given use case, such as depositing new images and metadata, annotating the images and depositing trained deep-learning models.

**Data ingestion**

Data ingestion is the process of adding new data to the system, encompassing tasks, such as crawling, parsing, validating and transforming information to be indexed. This process includes several data types, including metadata, images, annotations, analytics pipelines (which includes services and models) and so on. To this end, specific tools should handle incoming data to the infrastructure, following different paths depending on the data’s source and type.

When a new dataset is submitted, each entry undergoes a series of tasks to parse, validate and transform the information to facilitate a standardised entry. This may include crawling additional data from external services like Wikidata or to compute metrics, validate geographic coordinates and map them to locations. Additionally, this process will check for duplicate entries, based on the existing data in the infrastructure.

**Image annotations**

One of the key features of the system will be the ability to provide annotations for the existing images. When a set of annotations is supplied, these need to be ingested, validated and transformed into standard data types and structures, depending on the problem (e.g. classification, object detection, natural language processing and optical character recognition). After preprocessing, the set of annotations will be additionally validated to find whether they duplicate existing annotations, if the attached labels make sense, if the tagged region falls inside of the image and so on. This information will then be indexed and provided by the repository component and can be included in datasets, which will serve to improve existing inference tools and develop new ones.

**Machine-learning models and analytics services**

The same applies to other tasks, such as submitting a new analysis pipeline. New pipelines include data and metadata; machine-learning models; source code; service containers; automated workflow and service provisioning information as code; results and others. Each of these must be verified and tested, before being included as part of the analytics toolset.

An analytics pipeline sub-component comprising a set of services and functionalities is responsible for processing images or other media, to automatically infer information that would otherwise be manually tagged, for example, identifying a specific trait. To this end, each service provides specific functionality and comprises a sequence of instructions, from using multiple pre-trained models, to image transformations or other solutions, depending on the problem at hand. For instance, when ingesting a dataset, for each given specimen image, various analytics pipelines will be scheduled to run, each made of different steps and deep-learning models trained for specific tasks (e.g. detect mercuric chloride stains, identifying specific traits, extracting label information).

**Build machine-learning models and services**

Analytics pipelines are built of pre-trained models, as well as containerised applications and services previously built. The most computationally intensive part of the infrastructure will be training, building and updating these. It should be possible to schedule the execution of these heavy tasks, including data preparation (e.g. resize, augmentation), configuring the environment and parameters, training the models, assessing the performance and building, testing and packaging the services.

The system must allow the definition of service workflows as code, from the infrastructure, to model training and application packaging. This requires two parts. First, fully documenting modelling experiments to guarantee reproducibility, such that anyone can rerun the experiment and obtain the exact model and results. This involves the system indexing the data (i.e. link to the exact dataset) and code with the exact environment (e.g. by using conda and venv under Python or renv in R), the pre-trained models and all the required parameters, hyperparameters and similar, as well as controlling the randomness of such models (e.g. initialising seed state).

Secondly, the entire analytics pipeline should be documented as code, from infrastructure to application level. This allows for the exact replication of the build, test, package and deployment. Over the last decade, several technologies and sets of practices have appeared to attain such goals, normally linked to software development concepts, such as DevOps, MLOps and GitOps. GitHub provides Actions to attain continuous integration and deployment, allowing the automation of the entire workflow of a software service, from building to testing and deploying, based on simple text files (YAML). On the other hand, Docker images and similar solutions allow services to be containerised using similar simple definitions and shared across various environments, enhancing consistency and portability, while simplifying deployment and scaling processes. Going a step further, it is nowadays possible to define both the infrastructure and how services interact as code too (e.g. used under Docker compose or with Terraform and Kubernetes).

Such concepts must be exploited by the processing component, allowing submission of novel analytics pipelines. As the number of annotated datasets grows over time, the system might schedule the retraining of models and associated pipelines, reporting results and, if desired, replacing the existing analytics pipelines. Moreover, all the details, code and pre-trained models can be provided, so anyone can reuse them anywhere. Given the computation power needed, possibly requiring several GPUs for bursts of work, hybrid solutions offloading part of this work to cloud providers could be implemented, as an alternative to hosting and managing GPU clusters.

**Data integration**

Data integration will push the data generated by the above-mentioned sub-components to the respective parts of the system - the repository (e.g. metadata registry of the trained models and images, datasets, annotated data etc.) and the storage (e.g. image files and their derivatives, pre-trained models, metadata packages etc.).

**Data export**

The system will catalogue millions of specimens, each with variable amounts of metadata. These data can be filtered with complex queries, based on several parameters and fields. As an example, a user might want to search for records of a specific species, containing images and annotate them regarding the presence of signatures within a specific timespan. Requesting the generation of an image dataset, based on the result of such query, requires several processing tasks for scheduling, from the extraction and merging of the relevant metadata into the desired format, to resizing images if needed, assigning a persistent identifier, generating a dataset page and notifying the user. Moreover, if images and annotations for the same search criteria are updated in the following months, the user might request the dataset to be updated, generating a second version and assigning a new or versioned persistent identifier. Part of this functionality is already demonstrated by GBIF, which uses background jobs to export datasets on user request (excluding images and DOIs, but allowing the export of metadata, based on queries). Moreover, this sub-component may also be responsible for exporting machine-learning datasets to public platforms, such as the Registry of Open Data on AWS or Google Datasets, allowing users to easily mount them on external cloud solutions.

## Discussion

The 21^st^ century is already seeing catastrophic changes in global biodiversity. The resources needed to monitor and address these changes are far greater than the cadre of professional ecologists and taxonomists can provide. Machine learning promises to dramatically increase our collective capacity and, in complementary fashion, prioritise the attention of human taxonomists where it is most needed.

There are direct benefits of our envisaged infrastructure to biodiversity and research into artificial intelligence, but there are also positive impacts for society, the economy, the environment and for collection-holding institutions, for example, in support for more evidence-based environmental policy; improved pest detection and biosecurity; better monitoring of endangered species and better environmental forecasting to name just a few (also see Popov and Shevskaya (2021)).

Making images accessible in a common infrastructure is an opportunity for collections with limited resources to gain access to tools that would otherwise be unavailable to them. Indeed, Open Access for all researchers, including those from the Global South, is critical to ensure that collections fulfil their obligations to access and benefit sharing. As a large percentage of the world’s natural history specimens are housed in the Global North, scientists from the Global South are excluded from data on their own countries unless suitable access is provided (Dahdouh-Guebas et al. 2003, Fazey et al. 2005). To facilitate this will require a commitment to openness, ease of use, good tutorials, user-focused design and capacity building.

Such an infrastructure aligns with the European Strategy for Data (European Commission 2020), which aims to overcome challenges related to fragmentation, data availability and reuse, data quality and interoperability and dissolve barriers across sectors. Having a global infrastructure in place will incentivise natural history collections and their funders to digitise their specimens and attract funding to do so.

**Opportunity, obstacles and risks to realising a shared infrastructure for natural history collections**

Given the many use cases, the large number and diversity of stakeholders and the potential for innovative services and research, what is holding us back from creating the proposed infrastructure? One clear issue is that experts in machine learning are not always aware of the needs or potential of biological collections. These communities should be brought together to find the areas where collections can benefit from generalised approaches. A lack of standardisation and consequent lack of interoperability further impedes progress (Lannom et al. 2020). The Biodiversity Information Standards (TDWG) is just one of the organisations that might support development of such standards, notably the Audubon Core maintenance group, who maintain Audubon Core, a standard for the metadata of biodiversity multimedia resources (Morris et al. 2013). TDWG have worked in close collaboration with GBIF to develop standards on biodiversity and one could imagine similar alliances would benefit the envisaged infrastructure and its users.

We suggest that the most intractable obstacles to a shared, global infrastructure are socio-political. We envisage an infrastructure without institutional and national borders, in which people, organisations and nations are co-beneficiaries of a system, in which knowledge, skills, financing and other resourcing are acknowledged (Pearce et al. 2020). Furthermore, tracking the provenance of resources is also needed to ensure reproducibility and replicability of the system (Goodman et al. 2014).

Experiments so far lack scalability, often have manual bottlenecks and experience significant time lag in production of results due to limited access to computational and physical resources and to human resources to create and curate training datasets (Wäldchen and Mäder 2018).

The establishment of a new paradigm in research on collections impacts the frameworks and workflows currently used in collection curation and the research based on them and can, therefore, be disruptive. One of the greatest risks is introducing inherent errors and biases that are derived from the algorithms and prejudices that may be embedded unknowingly in training data (Boakes et al. 2010, Osoba and Welser 2017).

The institutions that hold collections have safeguarded this rich resource of information about biodiversity and natural history. They are major stakeholders for these materials to be preserved and associated data to become available for researchers and society. Paradoxically, making the data accessible digitally might create the illusion that there is no need to maintain the collections physically. In fact, the more information we can extract and link, the more valuable physical collections become for any future technology that can be applied to them. It is, therefore, critical to guarantee the link between the digital and physical specimen to ensure neither becomes obsolete, risking the real value attached to both.

**The future**

Objects in natural history collections represent one of the most important tools to understand life on our planet. Mobilising the capacity to analyse billions of objects with the help of machine learning is essential to meet the challenge of conserving and sustainably using biodiversity. This paper is written to emphasise the huge potential and the challenges. The main limitation to achieving our vision is not the software for machine learning, nor the ideas for using it, but the accessibility of data and images of specimens in a computational environment where they can be processed efficiently.

Many additional uses can be imagined for the analysis of non-specimen data, that is, the additional information that is linked to the physical object, either when directly written on attached labels or linked to inventories, catalogues or spreadsheets (Hardisty et al. 2022). There is also enormous potential for biological collections that have, so far, not been the main focus of digitisation, including microscope slides of thin sections; histological; or other extractions (e.g. Fig. 2b). Although imagination is the ultimate limit, we are currently limited by the availability of infrastructure to conduct such research.

## Figures and Tables

**Figure 2a.**
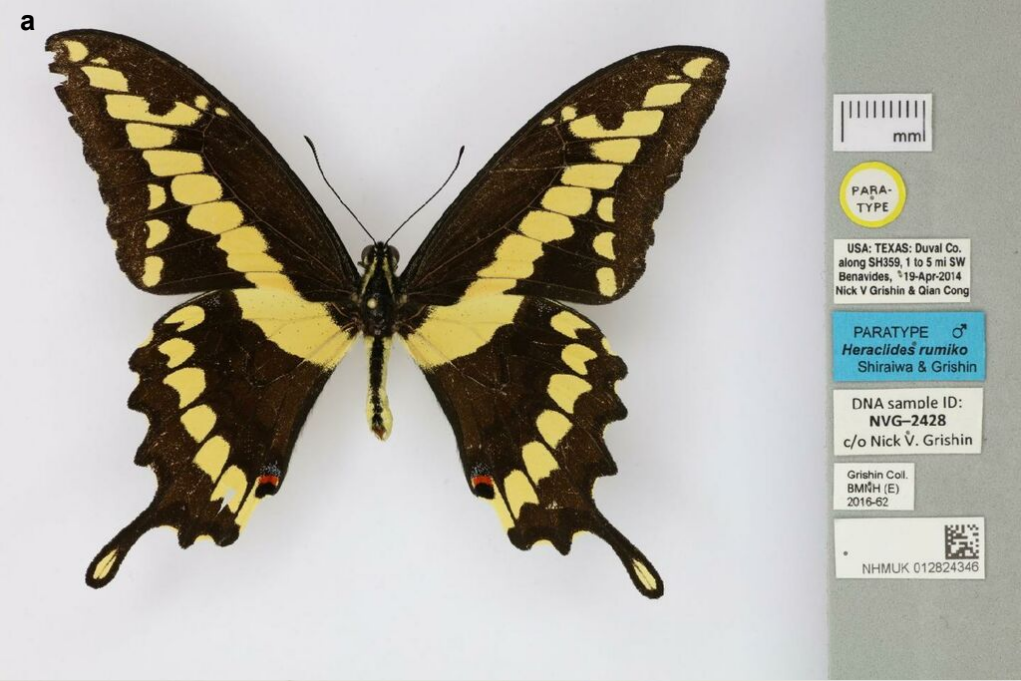
Paratype of *Heraclidesrumiko*, showing information encoded on multiple labels. Catalogue number NHMUK012824346 by The Trustees of the Natural History Museum, London (CC-BY).

**Figure 2b.**
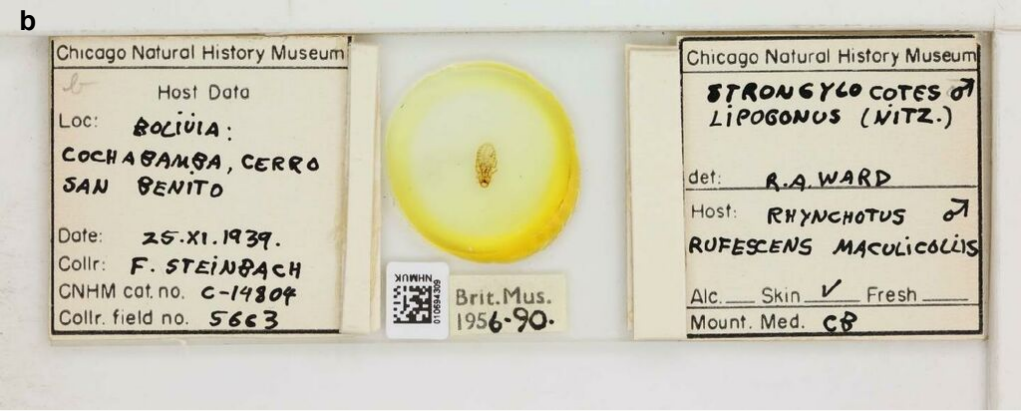
Specimen of a chewing lice (Philopteridae): *Strongylocoteslipogonus*, a parasitic species including host information on the label. Catalogue number NHMUK010694309 by The Trustees of the Natural History Museum, London (CC-BY).

**Figure 3a.**
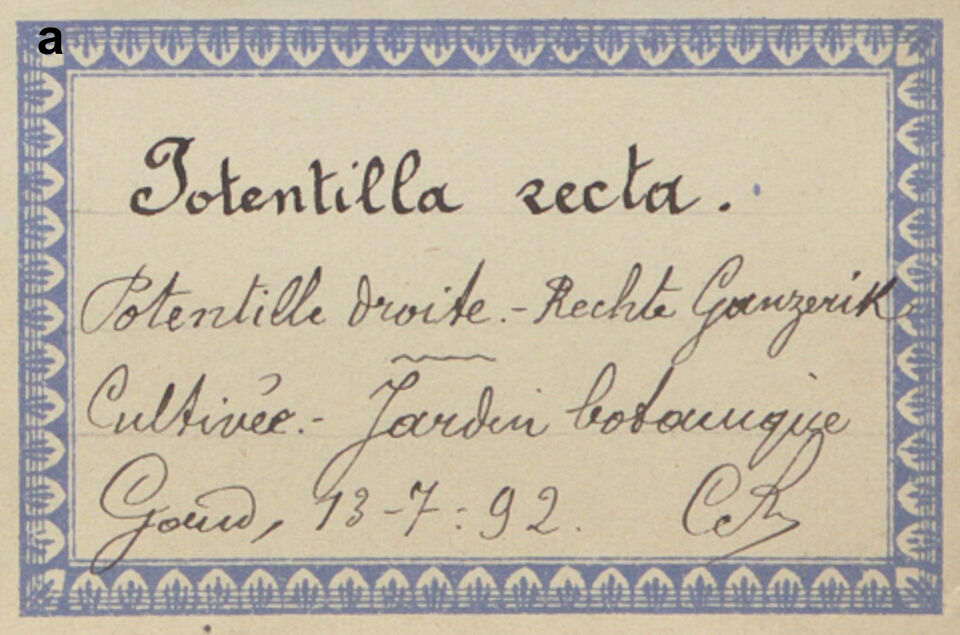
Label of *Potentillarecta* with distinctive label decorations (BR0000009398214; CC-By-SA) (B);

**Figure 3b.**
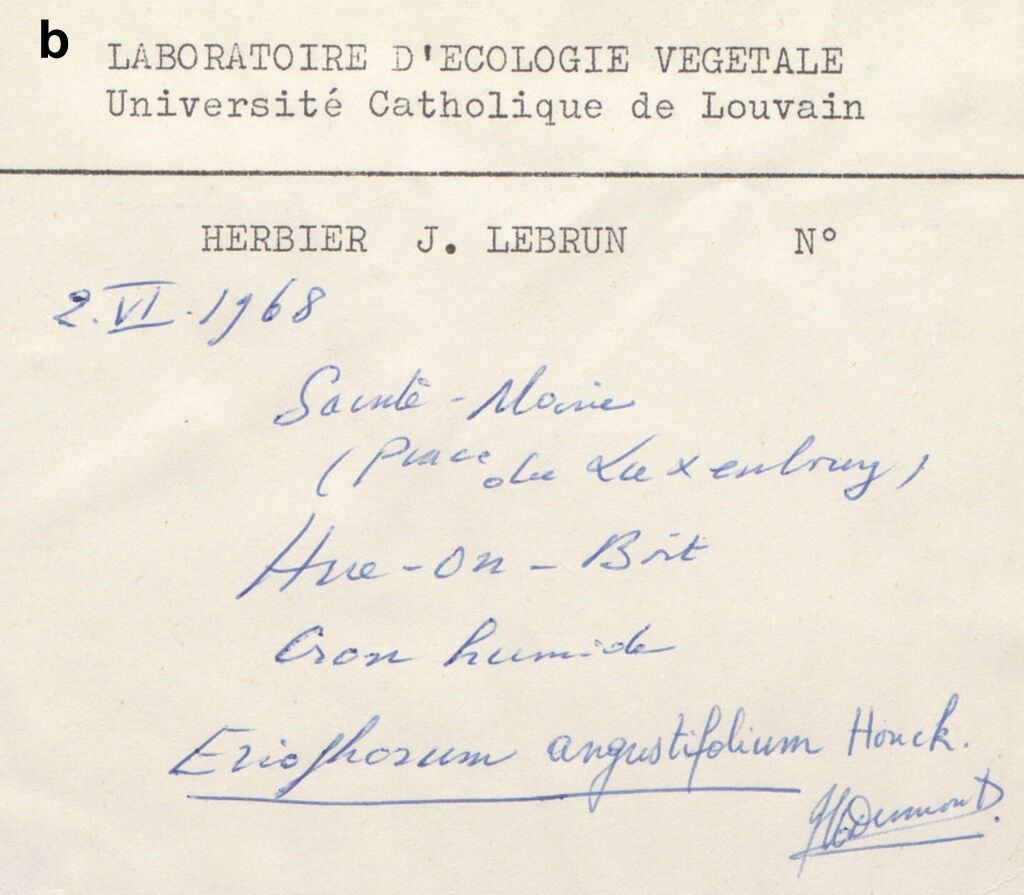
Label of *Eriophorumangustifolium* where collector’s signature can be recognised (BR0000005134137; CC-By-SA);

**Figure 3c.**
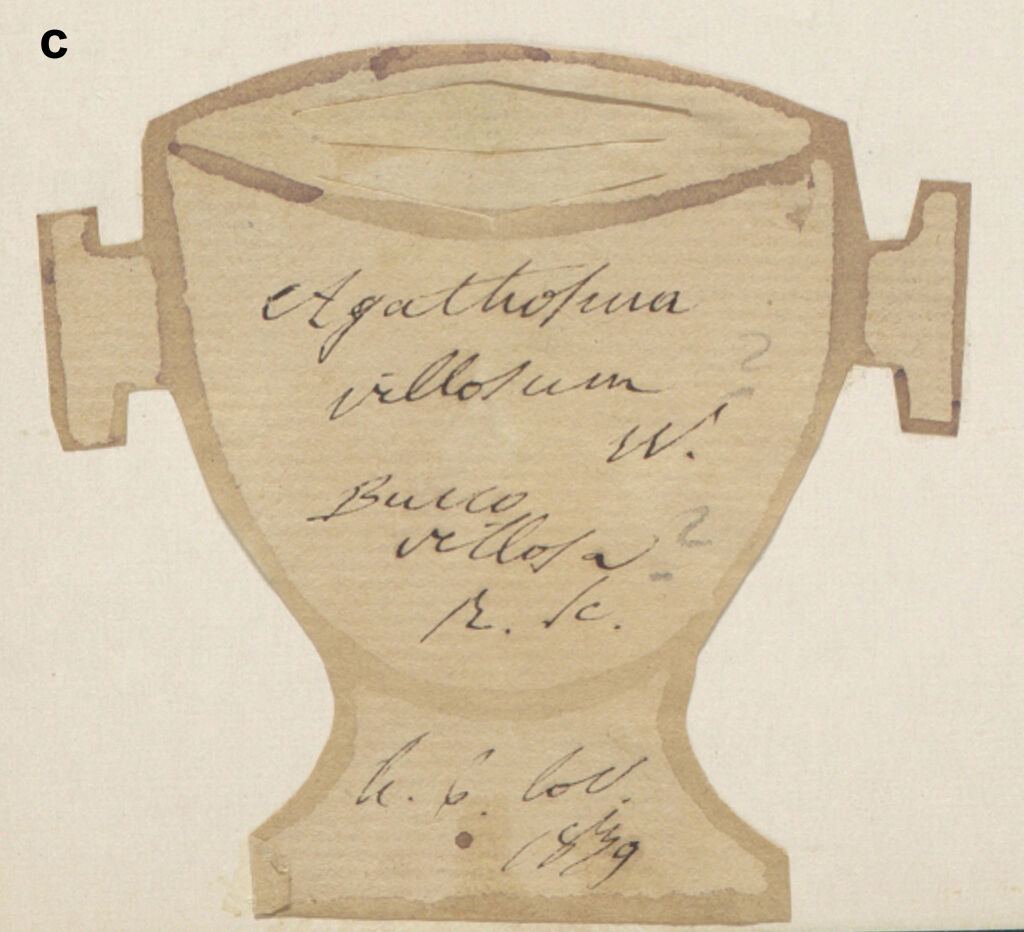
Distinct cup-shaped label of *Agathosmavillosum* (BR0000015671271; CC-By-SA);

**Figure 3d.**
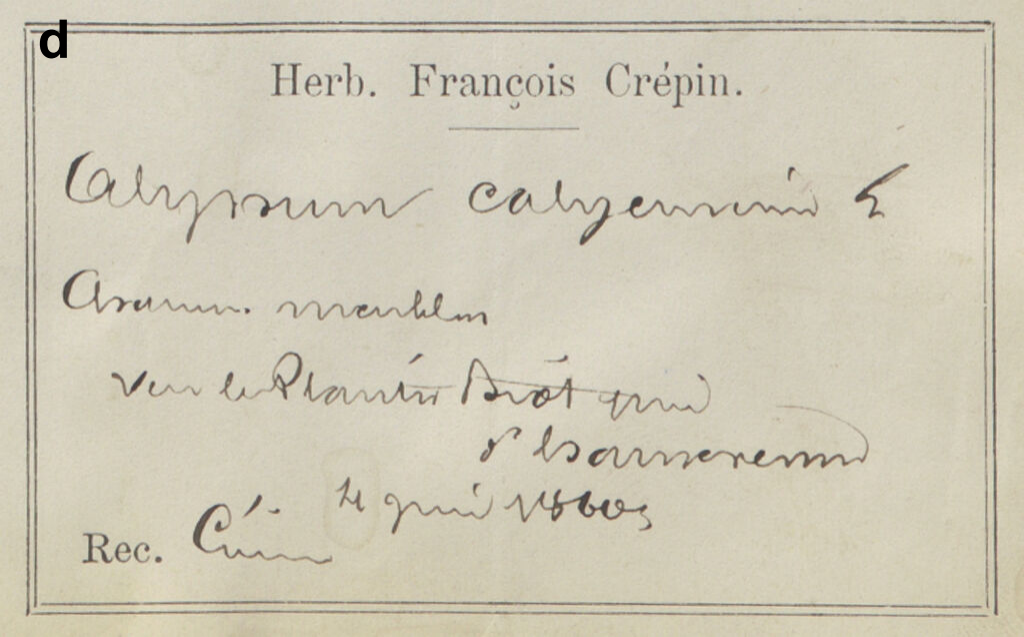
Label of *Alyssumcalycinum* collected by François Crépin, notorious for illegible, but recognisable handwriting (BR0000010426135; CC-By-SA).
